# Supplementary material for: Are carbon emissions trading and green financial instruments synergistic? -Comprehensive quantitative research based on content analysis
Source: PLoS One. 2024 Mar 7;19(3):e0298601. doi: 10.1371/journal.pone.0298601 (PMC10919601; doi:10.1371/journal.pone.0298601)
Supplement: S1 Data — (ZIP) [file pone.0298601.s001.zip › Data/地级市Z_F工作报告.zip!/╝¬┴╓/░╫╔╜╩╨/└┤╫╘╣1⁄2╓┌║┼í╛┬φ┐╦╩2╛▌═°í┐_2013.docx]

各位代表：

　　现在，我代表市政府向大会报告工作，请予审议，并请列席人员提出意见。

　　一、回顾过去五年：拼搏奋进，合力攻坚，白山转型彰显深刻变化

　　2008年以来，我们在省委、省政府和市委的正确领导下，在市人大、市政协的监督支持下，坚持以科学发展观为统领，以转型发展为主线，以富民强市为目标，以民生改善为根本出发点，紧紧抓住资源型城市经济转型试点市等政策机遇，团结带领全市各族人民，拼搏进取，务实创新，白山步入了历史上增长速度最快、发展质量最好、改革开放成果最丰、城乡面貌变化最大、人民群众得实惠最多的新阶段。

　　——过去五年，经济持续快速增长，综合实力显著提升，五年实现了“五大跨越”。坚持打资源牌、走特色路，科学发展、加快振兴，综合实力争先进位。一是总量扩张实现新跨越。预计2012年，实现地区生产总值654亿元，是2007年的2.8倍。固定资产投资550亿元，是2007年的2.6倍。全口径财政收入68亿元、地方级财政收入42亿元，分别是2007年的3.3倍和3.8倍。2011年我市综合增长竞争力在全国294个地级以上城市中位列第三。二是经济质量实现新跨越。“三五二”产业不断壮大，资源型工业比重比2007年下降5.7个百分点，高新技术和精深加工业提升5.9个百分点。以长白山国际度假区和亚洲顶级滑雪场为标志的旅游服务业异军突起，现代服务业、新兴产业投资比重提高到46.5%和21.3%。金刚水泥粉煤灰综合利用、华能煤矸石电厂等一批循环经济项目竣工投产，被列为全国餐厨废弃物资源化利用和无害化处理试点市。节能减排成效显著，单位GDP综合能耗下降22%，二氧化硫、化学需氧量削减10%。被评为全省“十一五”节能减排先进市。三是工业经济实现新跨越。预计2012年，实现规上工业增加值374亿元，是2007年的4.1倍。民营经济主营业务收入突破千亿元，是2007年的4.9倍。累计投资706亿元，实施技改项目1851项，开发新产品450种，新产品产值达到315.5亿元，年均增长48.8%。质量兴市向质量强市迈进，中国驰名商标达到7个、名牌产品4个，省著名商标69个、名牌产品52个，施慧达、喜丰聚氯乙烯果树专用膜荣获中国专利优秀奖，方大、露水河、地王、合兴被评为全省最受信赖、最具影响力、最具发展前景商标，人参、硅藻土、松花石3个省检中心落户白山。四是农村经济实现新跨越。预计2012年，实现农业总产值97亿元，比2007年增长86%。培育省级以上农业产业化企业44户，产业化产值140亿元，是2007年的2.8倍。退粮进特36万亩，高效作物比重超过60%，粮经菜比例由8:1:1调整到6:3:1。人参产业二次振兴，开发人参药食同源产品5大系列78个品种。特产业总产值72亿元，是2007年的2.3倍。县域经济实力明显提升，抚松县、江源区跻身全省十强。五是三产服务业实现新跨越。预计2012年，实现服务业增加值200亿元，是2007年的2.7倍。接待国内外游客500万人次，旅游社会总收入50亿元，分别是2007年的2.5倍和4倍。“万村千乡市场工程”连锁农家店突破1200户，实现乡（镇）村全覆盖。全社会消费品零售总额192亿元，是2007年的2.5倍。银行业本外币存贷款余额493.6亿元和311亿元，分别是2007年的2.2倍和1.8倍。新增保险经营机构5家，累计实现保费收入50亿元。担保公司达到9家，累计担保贷款89亿元。

　　——过去五年，改革开放呈现新格局，发展活力明显增强，五年取得了“四大突破”。坚持深化改革、扩大开放，内生动力明显增强。一是争取政策空间取得新突破。经不懈努力，资源型城市经济转型试点市政策延续到2015年，被国家确定为主体功能区规划重点生态区、第三批小城镇建设试点市和再生资源回收利用体系试点市，边境县份全部享受西部大开发政策，全市纳入长白山林区生态保护与经济转型规划，城区老工业区搬迁改造政策扎实推进。累计争取国家和省扶持项目1302项、各类补助和专项资金205亿元，居全省前列。累计财政支出500亿元，人均全省第一。二是改革创新取得新突破。95户厂办大集体企业完成改制，2.5万名职工全部解除劳动关系。政府机构改革、国库集中收付改革和集体林权制度改革主体任务完成。事业单位改革、农村综合配套改革扎实推进。率先与省科技厅、旅游局、工信厅、气象局、质监局等建立厅市会商机制。中国金属镁产业示范基地及全省镁产业、硅藻土产业技术创新联盟落户白山。三是对外开放取得新突破。坚持走出去、请进来，成功引进了华能、万达、山水等一大批战略投资者，实现引资向选资转变。预计2012年，全市实际引进市外资金506亿元，是2007年的5.2倍。实际利用外资1.77亿美元，是2007年的2.4倍。外贸进出口总额3.2亿美元，是2007年的2倍，对朝贸易突破8000万美元，是2007年的2.6倍。四是服务型政府建设取得新突破。行政电子监察系统实现省市县三级联网，审批项目减少到246项，行政许可提前办结率由36%提高到99.3%，是全省审批项目最少、时限最短的地区。自觉接受市人大、政协监督，办理人大代表建议和政协委员提案625件，办复率100%。深入开展服务型机关建设，政府效能不断提升。

　　——过去五年，承载功能大幅提升，城乡面貌日新月异，五年发生了“三大变化”。坚持完善配套、提升品位，发展环境明显优化。一是城市建设发生新变化。中心城区累计投入建设资金86亿元，超过前20年总和。新建城市道路98条71公里，实施了向江大桥、浑江干流堤防改造等126项工程。中心城区扩容升级，建成区扩大4.7平方公里。新增建筑面积450万平方米，相当于再造一个新城区，城市面貌换新颜。生态城镇化扎实推进，松江河镇、二道沟村等一批生态镇村通过国家级验收，靖宇镇等9个镇被列入全省“百镇建设工程”，抚松新城初具规模。全市城镇化率达到68.6%。二是交通建设发生新变化。累计投资146.6亿元，新增公路里程1268公里。营松高速辉南至靖宇花园口段贯通，高速公路实现零突破。新改建国省道549公里、农村公路679公里，实现乡乡通油（水泥）路，行政村通达率97%。宇松铁路开工建设，长白山机场开通5条航线，初步形成了以公路为主，铁路、航空为辅的立体交通体系。连续两年被省政府评为交通运输发展先进市。三是新农村建设发生新变化。累计投入建设资金43亿元，改造泥草房2.9万户、175.9万平方米，解决了17.3万农村人口安全饮水问题，256个示范村道路硬化、动力电入村、自来水入户均达到100%，村容村貌明显改观。

　　——过去五年，民生福祉显著改善，社会建设全面进步，五年迈上了“四大台阶”。不断加大投入，新增财力80%以上用于民生，比2007年提高20个百分点。一是城乡居民收入迈上新台阶。全市城镇居民人均可支配收入和农民人均纯收入达到21255元和8654元，分别是2007年的1.8倍和2倍，人均储蓄存款余额2.5万元，是2007年的2.1倍，居全省前列。二是社会保障体系建设迈上新台阶。五年新增城镇就业23万人，农村劳动力转移就业12.4万人，创建了长白山特产创业大市场等16个创业孵化基地，城镇登记失业率控制在4%以内。先后启动了“五七”家属工、新农保和城镇居民参保试点工作，养老、失业保险政策性覆盖面达到100%，基本医疗、工伤和生育保险覆盖率分别达到91.9%、77.5%和62.5%。城乡低保年年提标，应保尽保，由2007年的月人均130元和年人均693元分别提高到310元和1840元。“八路安居”工程完成954万平方米，“暖房子”工程改造729万平方米，被列为国家级重点示范市。三是社会服务功能迈上新台阶。累计实施民生实事100件，群众最关心、最直接、最现实的利益问题得到有效解决。教学条件进一步改善，教育质量明显提高，长白山职业技术学院填补我市高等教育空白。市中心医院和通矿总医院晋升三级医院，基层医疗机构全面实施基本药物制度，居民健康档案电子化率73.5%，新农合参合率99.7%。广播电视基本实现“村村通”。体育事业蓬勃发展，连续三年荣获全国群众体育先进单位称号。民族宗教工作得到加强，兴边富民行动和强基富民固边工程扎实推进，两次荣获全国双拥模范城称号。四是社会管理创新迈上新台阶。基层社会管理和服务体系试点建设全面启动。“五五”普法顺利完成，“六五”普法全面展开，法律服务水平明显提高。村民自治和城镇社区居民自治制度巩固加强。社会治安防控体系不断完善，命案侦破率连续四年全省领先。“一岗多责”安全生产机制进一步健全。战胜了2010年特大洪灾，市县防洪体系初步建立。实现连续33年无重大森林火灾。强化落实领导干部接访下访和包案制度，信访积案化解率95%以上，连续两年被省政府评为信访工作优秀单位。

　　即将过去的2012年，是本届政府的收官之年，也是精彩纷呈喜获丰收的一年。一是经济发展高速度。坚持稳增长、调结构、促发展，预计地区生产总值增长23%、固定资产投资增长30%、规上工业增加值增长25%，全口径财政收入、地方级财政收入、社会消费品零售总额均增长18%。财政支出150亿元，人均突破万元。主要经济指标综合排名全省前列。120万吨氧化球团、10万吨高纯二氧化硅、百万吨焦化、40万吨包装纸等一批牵动全局、影响未来的重大项目进展顺利。二是大事要事喜事多。全国最大旅游单体项目长白山国际度假区盛装开业，成功承办了首届中国民营经济发展（长白山）论坛、中国人参产业高峰论坛和全国汽车场地越野锦标赛，举办了长白山奇石暨松花石产品博览会，被命名为中国松花砚之都。成功签约总投资120亿元长白山广泽旅游度假区、总投资110亿元万达千万吨矿泉水、总投资100亿元长白山鹿鸣谷生态度假区等一批大项目，再次被评为中国最佳投资城市。三是攻坚破冰成果丰。经艰苦努力，白山至辉南高速公路批复建设，由“省地共建”调整为“省投省建”，极大降低了地方配套资金压力。省道白山至三道沟公路完成可研批复，争取上级资金3.8亿元。市区给排水系统纳入全省规划。争取资金9.2亿元，采煤沉陷区补充安置工程全面开工建设，解决了1万余户原A、B级受损户多年遗留问题。苏州商贸城、福泰大厦、鼎元林纸等滞缓工程取得突破性进展。四是共享发展惠民生。万亩蔬菜基地建设全面启动，建成后市区蔬菜自给量提高50%以上。争取上级资金8000万元，矿泉湖二水源建设加快推进。中心城区建设投资20.1亿元，是历史上最多的一年，新建改造城市道路24条、人行步道16万平方米，通江路、站东街等5条街路及浑江大桥通车，亮化主街路公建楼体35栋，“暖房子”及保障性安居工程完成400万平方米。九大民生工程33件民生实事如期完成，与此同时积极筹措资金，修建了通林路、东华街、站南街和七道江大桥，改造了通沟铁路道口，下大气力开展燃气安全大检查、火车站周边环境整治，百姓合理诉求得到有效解决。缉枪治爆专项行动全省第一，群众安全感满意度全省第三。全市政治安定、社会稳定，人民群众安居乐业。

　　五年来，统计、审计、水文、气象、人防、民兵预备役、地震、外事、地方志、档案、老龄、残联、妇女儿童等工作都为全市经济社会发展作出了积极的贡献。

　　各位代表，五年来，我们坚持科学发展，不断深化对发展规律的认识，在实践中走出了一条资源型城市科学发展、加速崛起之路，谱写了白山发展史上的绚丽篇章，经验弥足珍贵。这一时期坚定的发展思路是：坚持生态立市、产业强市，打资源牌、走特色路，全力构建“三五二”产业体系，推进“三化”统筹；这一时期强劲的发展动力是：坚持解放思想、改革开放，大力实施“三动”战略，敢为人先，有所为有所不为，着力释放全社会发展活力；这一时期浓厚的发展氛围是：坚持发展为先，努力做到不动摇、不懈怠、不折腾，聚精会神搞建设，一心一意谋发展，以事业鼓舞人心、以实干凝聚人心，使跨越发展成为全市工作的主旋律和最强音；这一时期根本的发展方法是：坚持正确的政绩观，谋划工作重长远，推动工作重细节，落实工作重成效，大力推进政府效能建设，不断提升工作水平。五年来的奋斗实践，不仅为推进白山跨越发展奠定了坚实基础，进一步坚定了我们率先崛起的信心和决心，也为未来白山实现跨越发展积累了宝贵财富。

　　各位代表，五年的发展历程波澜壮阔，五年的成就让我们倍感自豪。成就来之不易，发展振奋人心！这是省委、省政府和市委正确领导的结果，是市人大、市政协和社会各界有效监督、鼎力支持的结果，是全市各族人民和各行各业群策群力、拼搏实干的结果。在此，我代表市政府，向全市各族人民，向驻白山人民解放军指战员、武警部队官兵、公安司法干警、中省直单位，向各民主党派、工商联、人民团体、无党派人士、离退休老同志，向关心支持白山发展的各界朋友，致以崇高的敬意和衷心的感谢！

　　在看到成绩的同时，我们也清醒地认识到，前进中还存在不少困难和问题。主要是，经济总量不大、结构不优的基本市情仍未根本改变，经济转型任重道远；城市基础设施历史欠账多，管理机制亟待创新；改革和发展中利益交织的各种矛盾沉淀较多，维稳压力大；政府职能和工作作风转变仍需加强，社会管理和公共服务有待于进一步创新。对此，我们务必高度重视，在今后工作中认真加以解决。

　　二、展望未来五年：戮力同心，奋勇争先，全力建设全省较发达城市

　　未来五年，是全面完成“十二五”规划各项任务目标的关键时期，是深化改革开放、实现转型发展的攻坚时期，也是我市加快振兴、加速崛起的决战时期。尽管国际经济形势严峻复杂，国内经济运行面临诸多挑战，我市经济社会发展不确定因素增多，但经过多年努力，我们已具备跨越发展的坚实基础和再上新台阶的有利条件，特别是东北边境地区享受西部大开发政策、长白山林区生态保护与经济转型上升为国家战略，为我市全面赶超带来了新的重大机遇。我们必须增强机遇意识、赶超意识，在新一轮发展中抢占先机，赢得主动，奋力在全省争先进位、在东北东部率先崛起。

　　根据市六次党代会部署，未来五年政府工作的总体要求是：高举中国特色社会主义伟大旗帜，以邓小平理论、“三个代表”重要思想和科学发展观为指导，深入贯彻落实党的“十八大”精神，坚持生态立市、产业强市，打资源牌、走特色路不动摇，突出“发展”和“民生”两个关键，实施“三动”战略，推进“四化”同步，建设生态文明美丽新白山，建成全省较发达城市、全国最平安城市和最稳定边境地区，全面建设小康社会，让城乡居民生活得更加美好。

　　未来五年主要奋斗目标是：努力实现“四翻番、双倍增、两领先、一跃升”。“四翻番”，即到2017年，全市地区生产总值、固定资产投资、全口径和地方级财政收入在2012年的基础上翻一番以上，分别达到1600亿元、1650亿元、190亿元和110亿元以上；“双倍增”，即全市城镇居民人均可支配收入和农民人均纯收入在2012年的基础上增加一倍以上，分别达到4.3万元和1.8万元以上；“两领先”，即主要指标增速和人均指标继续全省领先；“一跃升”，即三次产业结构调整到7:53:40，三产服务业比重提升8.8个百分点。

　　——坚持以特色鲜明、转型升级、产业发达为目标，全力打造实力新白山

　　打响“三大名城”和“三大基地”金字招牌，大力实施“333”产业集群梯次推进计划，培育1000亿元、300亿元、100亿元以上三个梯次产业集群，增加值占GDP比重80%以上。

　　第一，培育三个千亿级产业集群。一是能源产业集群。延伸煤炭产业链条，扩大火电、发展水电，开发风力、生物质、光伏发电，加速白山油气田勘查。到2017年，全市煤炭及煤化工产品达到2600万吨，电力装机300万千瓦。能源产业产值达到1000亿元以上。二是矿泉饮品产业集群。坚持打品牌、上层次、扩总量、控资源，推进万达等一批矿泉饮品项目，大力开发中高端系列化产品。到2017年，全市矿泉饮品产能达到2000万吨。矿泉饮品产业产值达到1000亿元以上。三是矿产冶金新材料产业集群。扎实开展找矿突破战略行动，建立重要矿产资源战略接续区，全力推进整装勘查和精深加工。到2017年，全市铁矿石产量增加到1600万吨，铁精粉、氧化球团、硅藻土、镁合金和黄金产量分别达到500万吨、370万吨、75万吨、40万吨和6吨。矿产冶金新材料产业产值达到1000亿元以上。

　　第二，培育三个三百亿级产业集群。一是旅游文化产业集群。打造5A级长白山国际度假区，建设松江河旅游经济圈和松花江、鸭绿江旅游经济带。深度挖掘长白山文化内涵，打造一批人文底蕴深厚、地域特色鲜明的文化旅游新卖点。建设长白山旅游集散中心、东北亚旅游名城和国际旅游目的地。到2017年，全市接待游客1400万人次。旅游文化产业总收入300亿元以上。二是人参产业集群。扩大人参标准化种植面积，大力开发药食同源产品，打造龙头企业和知名品牌。到2017年，全市人参、西洋参产量稳定在1200万公斤，精深加工率70%以上。人参产业产值达到300亿元以上。三是林产品产业集群。壮大金隆、丽美坚、和平等一批龙头企业，打响地王地板、露水河刨花板、恒和家具等驰名品牌。到2017年，全市复合地板产能2700万平方米、人造板150万立方米、家具50万件套。林产品产业产值达到300亿元以上。

　　第三，培育三个百亿级产业集群。一是医药产业集群。大力扶持以修正、神威为代表的中药现代化企业、以施慧达为代表的化药和生物药企业，推动医药产业快速发展。到2017年，全市中药材种植面积达到25万亩。医药产业产值达到100亿元以上。二是特色农产品产业集群。坚持“退粮进特”不动摇，着力发展绿色、有机、无公害现代特色农业。到2017年，全市高效作物比重达到70%以上，粮经菜比例调整到2:6:2。特产业产值达到140亿元以上。三是现代物流产业集群。建成广泽购物中心、欧亚商都等核心商业区，打造靖宇内陆港和长白、临江区域性国际物流区。到2017年，全市货运量达到8000万吨。现代物流业产值达到100亿元以上。

　　——坚持以功能完善、品位提升、环境优化为目标，全力打造魅力新白山

　　优化空间布局，彰显城市特色，建设休闲养生、宜居宜业的国际生态旅游城。

　　第一，优化布局建设城市。坚持高起点、高标准规划城市，加快浑江沿岸发展轴及江北新行政中心、铁南新城副中心、老城区市级商业中心改造升级，构建中心城区“一轴三心”空间结构，推进基础设施、产业布局、市场体系、社会事业、户籍制度市区一体化发展，打造60万人口区域中心城市。建成县（市、区）行政所在地区域性中小城市和一批特色乡镇。推动商贸、物流、金融等产业融合互动，打造各类要素区域性服务中心，推进基础设施和公共服务城乡均等化。到2017年，城镇化率达到78%以上。

　　第二，优化区位发展交通。构建高效快捷的立体交通体系，建成营松、鹤大、白山至辉南高速，推进白山至临江、松江河至长白高速，建成宇松、松江河至长白、泉阳至白山镇铁路，积极争取城际及长白山高铁项目。全市新增高速公路360公里、铁路260公里，实现市通高铁、县县通高速和铁路。

　　第三，优化环境扩大开放。深化行政管理体制改革，积极稳妥推进事业单位改革，加快重点领域和关键环节改革。大力发展要素市场，营造公平竞争环境。强化自主创新体系建设，加快科技成果转化。积极对接长吉图、环渤海经济带，主动融入东北东部区域合作，打造鸭绿江上游开发开放先导区，深化与东北亚地区投资贸易合作，聚焦世界目光，在更高层次、更广领域实现大开放。

　　——坚持以资源节约、生态良好、绿色发展为目标，全力打造生态新白山

　　坚持生态文明建设长期主导战略，生态立市、节约优先、保护优先，着力推进绿色发展、循环发展、低碳发展、安全发展，建设天蓝、地绿、水净的美丽白山。

　　第一，实施环境质量提升工程。加快城镇污水、垃圾处理设施建设，加大松花江、鸭绿江、浑江干流水污染防治力度，强化大气、土壤污染防治，大力推进节能减排。健全生态环境保护责任追究和损害赔偿制度，增强全民节约意识、环保意识、生态意识。到2017年，全市城镇生活污水和垃圾无害化处理率80%以上，单位GDP综合能耗降低15%以上，主要污染物排放总量降低10%以上，松花江出境水质保持国家2类水体标准，鸭绿江、浑江干流达到国家3类水体标准，市中心城区空气优良级别天数340天以上。

　　第二，实施生态修复工程。全面落实“三北”防护林五期和第二个“十年绿化美化吉林大地”造林工程，全市森林覆盖率保持83.2%以上。加强鸭绿江、松花江生态沟系和小流域综合治理，完成浑江流域和曲家营水库上游生态修复工程，推进土地整理和复垦复绿。严格矿产开发准入，鼓励整装勘查、集约开发，禁止小规模、低水平重复建设。

　　第三，实施资源综合利用工程。引导资金、技术、人才向循环经济园区和企业聚集，建立政府主导、企业主体、公众参与、法律规范、科技支撑的循环经济发展机制，加快形成低消耗、低排放、高效率的生产模式。到2017年，全市工业固体废弃物综合利用率75%以上。

　　——坚持以社会文明、公平正义、人民幸福为目标，全力打造和谐新白山

　　坚持以人为本，加大民生投入，完善公共服务保障体系，努力做到民有其业、民有其得、民有其保、民有其居、民有其安、民有其乐。

　　第一，人民生活更加幸福。五年新增城镇就业22万人，实现更高质量就业。城镇居民养老、失业、基本医疗、工伤、生育保险和新型农村养老保险实现全覆盖，多层次弱势群体社会救助体系更加完善，实施保障性安居工程确保中低收入家庭住有所居，城乡居民收入与经济发展同步增长，人民群众生活水平和幸福指数明显提高。

　　第二，社会事业长足发展。教育事业优先发展，素质教育全面加强。医疗服务、公共卫生体系更加健全。实施文化强市工程，推进文化事业大繁荣。大力发展体育事业，保持群众体育和竞技体育全省优势地位。充分发挥广播电视宣传作用，营造良好的舆论氛围。提高出生人口素质，促进人口长期均衡发展。全面贯彻党的民族宗教政策，促进少数民族地区经济社会发展。加强精神文明建设，进一步提高群众文明素质和城市文明程度。

　　第三，社会管理显著加强。加强基层社会管理和服务体系建设，增强城乡社区服务功能。深化“六五”普法和依法治市，维护公平正义。完善信访制度，建立健全重大决策社会稳定风险评估机制，构建立体化社会治安防控体系，打造全国最平安城市。深入开展双拥共建活动，完善创新党政军警民合力治边机制，打造全国最稳定边境地区。全力抓好安全生产，确保人民生命财产安全，让人民群众安居乐业。

　　各位代表！经过未来五年的不懈努力，必将把白山建成生态环境更加优美、经济文化更加繁荣、发展活力更加强劲、社会秩序更加和谐的全省较发达城市，人民群众一定会生活得更加富裕、更加幸福！

　　三、2013年工作安排：重点突破，全力作为，实现政府工作良好开局

　　2013年是新一届政府的开局之年，做好明年工作事关未来五年发展。面对错综复杂、快速变化的形势，我们既要正视困难和挑战，又要看到有利条件和积极因素。当前，世界经济已由危机前的快速发展期进入深度转型调整期，我国经济社会发展基本面长期趋好，面临扩大内需、提高创新能力、促进经济发展方式转变的新机遇。为此，要继续把握好稳中求进的工作总基调，以提高经济增长质量和效益为中心，立足全局，突出重点，开拓创新，扎实开局。综合各方面因素，明年主要预期目标是：全市地区生产总值增长22%，全社会固定资产投资增长25%，全口径财政收入、地方级财政收入均增长18%，城镇居民人均可支配收入、农民人均纯收入分别增长15%和17%，城镇登记失业率控制在4.4%以内，单位GDP综合能耗降低3.5%。实现上述目标，要突出抓好八个方面工作：

　　（一）扩张总量，在项目建设上追求更大作为。抢抓政策叠加机遇，投资拉动扩大增量，产业升级盘活存量，全力谋划推进产业链条长、带动能力强的大项目、好项目。全年计划安排500个3000万元以上重点项目。一是竣工投产一批。抓好配套建设，加快工程验收，确保长白山国际度假区二期、喜丰农用塑料节水器材等100个项目年内竣工投产。二是加快建设一批。抢时间、保进度，加快推进东北亚铁路集团300万吨铁精粉、舜茂10万吨高纯二氧化硅等200个项目建设。三是争取开工一批。落实投资主体，完善前期手续，争取2个200万吨矿泉水、长白山广泽旅游度假区、白山至辉南高速等200个项目开工建设。四是谋划储备一批。研透国家政策导向，把握国内外资本流向，结合白山资源禀赋、产业布局，提高项目谋划的科学性和前瞻性，谋划80个亿元以上项目，其中10亿元以上30个、30亿元以上10个。五是整装勘查一批。大力开展找矿突破战略推进年活动，坚持风险勘查、资本技术结合、探采一体化 “三优先”原则，投资3.3亿元，实施整装勘查项目40个，力争取得一批重大勘查成果。

　　（二）提速增效，在工业经济上谋求更大突破。加强扶持引导，打好工业经济稳增长攻坚战，实现规上工业增加值464亿元。一是实施“双百”工程。推进“百名经济管理干部包保百户重点企业”活动，派驻专人“零距离”包保，引导企业内部挖潜、开拓市场、释放产能；扎实推进“百名县处级领导干部包保百户小微企业”活动，给政策，定任务、定目标、定措施包保，促其快速成长。二是实施企业成长工程。坚持提质量、促增量，引导扶持企业科技创新、管理创新、人才引进。加快建设科技创新公共服务体系，组织建立产业技术创新联盟，鼓励施慧达药业、喜丰塑料等重点企业建立产学研联合技术开发中心，引进新技术、开发新产品、创立新品牌。支持东锋镁业、琦祥纸业、长富远通加快上市步伐，积极培育后备上市资源。三是实施开发区“二次创业”工程。进一步推进开发区体制机制创新，在完善要素保障、提升配套功能、强化支撑带动上狠下功夫，坚持市县（区）一体化发展，全力打造长白山天然矿泉水、煤化工工业园等15个特色园区，加快开发区提档晋级。

　　（三）退粮进特，在农村经济上创造更高效益。坚持抓特色、调结构、增收入，实现农业总产值111亿元。一是优化结构强产业。大力发展参、蛙、菌、药、菜、牧、果特色产业，引导优势产业实现基地化生产、市场化经营、规模化发展。建设60个千亩退粮进特示范基地，全市退粮进特15万亩，人参GAP种植面积达到2000万平方米，林下参50万亩，专业乡镇和专业村15个和70个，特产业产值86亿元，增长20%。二是扶优促强壮龙头。坚持以工业化提升农业，以产业化致富农民。加快建设抚松、靖宇、长白三大人参产业园区，扶持大山合、林源春等龙头企业做大做强，推进林村中药、广药集团等重点产业化项目建设，进一步提高农产品精深加工能力和产业化水平。全市农业产业化产值达到170亿元，增长20%。三是强基固本建新村。坚持规划先行、试点示范、产业支撑，加快城乡一体化发展。投资8亿元建设450个新农村项目，高标准完成20个示范村、20个整体推进村建设和100个村“四清四改”任务，解决6.7万人口安全饮水问题，建设和谐新农村。

　　（四）兴旅活商，在三产服务业上实现更大提升。大力实施现代服务业跨越计划，打造精品，优化服务，提高层次。一是大力发展旅游产业。加快推进长白山广泽旅游度假区、东北亚国际论坛会议中心等项目建设，开辟松花江白龙湾、鸭绿江苇沙河水上游和长白山国际度假区至长白、临江自驾游线路。深入推进旅游环境综合整治，增强产业承载功能，提高服务水平和贡献率。全年接待国内外游客650万人次，旅游业社会总收入70亿元，分别增长30%和40%。二是加快发展流通服务业。加快广泽购物中心、欧亚商都建设，大力发展连锁店、电子商务等新兴服务业态。加快推进长白山国际度假区农产品物流园区、长白山特产城建设，建立完善现代商品流通体系。鼓励银行业加大金融服务力度，拓宽小微企业融资渠道。推进无线城市、智慧城市建设，提升信息化程度。

　　（五）招大引强，在对外开放上迈出更大步伐。不断创新招商方式，主动承接产业转移，积极引进战略投资。全年引进市外资金632亿元，实际利用外资2亿美元。一是全面深化区域合作。高质量办好第二届中国民营经济发展（长白山）论坛和第五届东北东部（12+2）城市区域合作圆桌会议，进一步加强与东北东部城市在旅游、物流等方面合作。长白口岸通过国家一类验收，争取设立长白国家级重点开发开放试验区。鼓励扶持中矿国际、曙光凯旋等企业开展对朝经济合作，引导丽美坚、华枫、金隆木业等企业海外投资。推进与东北亚地区在投资贸易、现代农业、现代物流、劳务输出等领域合作。二是全面扩大招商引资。强化招商奖惩机制，瞄准高新技术、现代物流、金融服务、旅游文化等重点领域，欧美、日韩、港澳台、环渤海、长三角等重点地区，国际国内500强、行业龙头等重点企业，搭借东博会、民营经济论坛等会展平台，以优质资源和优势项目引进战略投资者。切实提高招商项目履约率和开工率。三是全面优化投资环境。深化行政审批制度改革，搭建多部门联动汇审平台，进一步减少审批环节、压缩审批时限。大力倡导诚实守信，建立健全社会信用体系。整顿和规范市场经济秩序，促进市场主体守法经营。加强中介组织建设，发挥行业协会作用，促进行业自律。

　　（六）开源节流，在财税金融上取得更新成果。坚持加快发展保增长，增收节支调结构，深化改革增效益，集中财力办大事。一是加强税收征管。继续搞好税源普查，建立税源管控信息网络平台，加强对煤炭、矿产、矿泉饮品、木制品、房地产等重点税种、行业、企业的监管。加强非税收入管理，增加政府可用财力。二是优化支出结构。坚持保民生、保运转、保发展、保重点，向教育、医疗卫生、就业和社会保障、社会救助、基础设施建设等方面倾斜。加强对政府性投资项目和财政专项资金的管理，保证项目建设和资金使用安全高效运行。加强预算管理，严格执行“收支两条线”，大力压缩弹性支出，降低行政成本。三是强化金融保障。大力发展农村商业银行、村镇银行、资金互助社等地方法人金融机构，积极引进域外金融机构在我市设立分支，力争全市信贷增长高于全省平均水平。进一步完善中小企业信用担保体系，争取再组建1家亿元以上民营担保公司。创新金融服务方式，利用中小企业集合债、短期融资券、融资租赁、信托等多种形式为企业融资创造条件。

　　（七）建管并重，在城乡环境上取得更大进步。坚持提升规划、加快建设、完善功能、打造亮点、强化管理、注重经营，进一步优化人居环境。一是建设提速度。新建续建长白山大街西段、朝阳路等21条街路，配套完善雨污水系统。加快行政中心新区、金河大桥、浑江大街改造、矿泉湖二水源、月牙河及碱厂沟河治理等工程建设步伐。投资43亿元，开工建设白山至辉南高速，推进鹤大、营松高速和长松岭隧道、白山至三道沟公路建设，实现村村通油（水泥）路。推进保障性安居工程，努力扩大投资，促进房地产业健康发展。二是管理见成效。坚持提高市民素质和强化综合执法双管齐下，集中整治与长效管理有机结合，积极探索创新市区联管共建、市场运作融资等管理模式，进一步下移重心，将管理触角延伸至街道社区。突出抓好市中心区重点地段和窗口部位、县（市、区）行政所在地以及交通沿线环境治理。从严整治违法建设、违规用地等突出问题，优化配置土地资源。三是环境上档次。大力实施规划造绿、补植增绿、见缝插绿立体绿化。加大居民小区环境综合整治力度，加快推进城市亮化、老旧建筑立面和主街路牌匾改造。完成松花江等5个重点流域水污染防治规划，大力推进市生活垃圾处理场改扩建、白山热电机组脱硝等环保工程，中心城区和主要城镇全部达到环境空气质量二级标准，集中式饮用水源水质达标率100%。

　　（八）改善民生，在和谐创建上树立更高目标。全力办好八大民生工程40件民生实事，加快发展社会事业，让人民群众生活得更有尊严、更加幸福。一是加快发展科教文体事业，提高潜力指数。实施教育质量提升工程，加快长白山职业技术学院改扩建和县级职教中心实训基地建设。大力发展公益性文化事业，丰富群众文化生活。深化同高校院所在硅藻土、镁合金等产业领域的合作，搭建“政产学研用”合作平台。推动群众体育、竞技体育和体育产业协调发展，全力备战第十七届省运会

　　和省社区体育健身大会，办好2013全国汽车场地越野锦标赛。二是着重抓好卫生计生工作，提高健康指数。全面落实公共卫生均等化服务项目，完善基本药物制度和基层医疗卫生机构运行新机制，重点建设县域医疗卫生体系，县级医院全部达到二甲标准。健全新农合制度，推动支付方式改革，提高重大疾病保障水平。推进优生促进工程，提供生殖健康服务10万人次以上。三是全面提升社会保障水平，提高和谐指数。不断提高五大险种覆盖面，力争实现全民社保。城镇新增就业4.5万人，农村劳动力转移就业2万人。建立城乡低保标准与物价水平联动机制，完善社会救助制度。棚户区改造、采煤沉陷区补充工程及廉租房新建续建145.9万平方米。四是切实维护和谐稳定局面，提高平安指数。保持高压严打态势，命案现案破案率全省领先。积极推广“一站式接待、一条龙办理、一揽子解决”工作机制，力争信访案件结案率95%以上。加强食品药品安全监管。强化重点行业和领域安全隐患监管整治，坚决遏制重特大事故发生，实现连续34年无重大森林火灾。进一步完善党政军警民合力治边长效机制，大力实施兴边富民行动、爱民固边战略，全力推进边境视频监控网试点工作，切实维护边境地区和谐稳定。

　　四、政府自身建设：务实重行，奋发有为，努力提升各项工作新水平

　　新的形势和任务对政府工作提出了更新更高的要求，必须全面加强政府自身建设，努力打造为民、务实、廉洁、高效政府。

　　（一）坚持与时俱进，提高学习力，努力建设创新政府。开放视野，博采众长，学以致用，以学增智、以学明理、以学修身，打造学习型政府、研究型队伍。坚持在实践中求创新、发展中谋创新，围绕加快经济转型、统筹推进“三化”、构建“三五二”产业体系、强化社会管理等重大问题，开拓创新，科学谋划，提能善政，推行目标化、项目化管理工作法，做到重程序、重细节、重成效，确保各项工作统筹高效推进。

　　（二）坚持依法行政，增强约束力，努力建设法治政府。严格按照法定权限和程序行使权力、履行职责，做到规范执法、公正执法、文明执法。进一步完善议事规则和决策程序，做到民主决策、科学决策、依法决策、审慎决策，提高决策质量。严格执行向人大及其常委会报告工作和人民政协通报情况制度，自觉接受人大工作监督、法律监督，政协民主监督和社会监督。认真负责办理人大代表建议和政协委员提案，确保办复率，提高满意率。

　　（三）坚持以人为本，增强凝聚力，努力建设服务政府。扎实落实中央密切联系群众八项规定，进一步改进工作作风，坚持民生优先、服务为先，深入基层调研，总结经验、研究问题、解决困难、指导工作。广纳群言，广集民智，悉心解决群众的合理诉求，坚决打击损害群众利益的突出问题，切实保障群众的合法权益，维护社会公平正义，做到言必信、行必果。

　　（四）坚持真抓实干，提高执行力，努力建设高效政府。认真开展批评和自我批评，勇于自我革新，强化责任担当，敢于动真碰硬。大力发扬脚踏实地、埋头苦干、雷厉风行、重行践诺作风，转会风、改文风，说实话、办实事，出实招、求实效。推行一线工作法，做到干部工作在一线，政策落实在一线，问题解决在一线，作风转变在一线，成效检验在一线。强化行政问责和绩效管理，奖优罚劣，确保政令畅通。

　　（五）坚持从严治政，提升公信力，努力建设廉洁政府。深入推进教育、制度、监督并重的惩治和预防腐败体系建设，从源头上、制度上规范行政行为。坚持纠建并举，切实纠正部门和行业不正之风。加强执法监察、廉政监察、效能监察，强化审计监督，严肃查处各类违法违纪行为。认真落实工作责任制和责任追究制，厉行勤俭节约，严格遵守廉洁从政有关规定，做到有权必有责、用权受监督、违法要追究，树立清正廉洁政府形象。

　　各位代表！新的目标催人奋进，新的征程任重道远。让我们以党的十八大精神为指引，在市委的坚强领导下，抢抓机遇，开拓创新，心无旁骛，攻坚克难，为实现全省较发达城市的宏伟目标而努力奋斗！
